# Self-assembled PEG-*b*-PDPA-*b*-PGEM copolymer nanoparticles as protein antigen delivery vehicles to dendritic cells: preparation, characterization and cellular uptake

**DOI:** 10.1093/rb/rbw044

**Published:** 2017-02

**Authors:** Pan Li, Junhui Zhou, Pingsheng Huang, Chuangnian Zhang, Weiwei Wang, Chen Li, Deling Kong

**Affiliations:** 1 Tianjin Key Laboratory of Biomaterial Research, Institute of Biomedical Engineering, Chinese Academy of Medical Sciences and Peking Union Medical College, Tianjin 300192, China; 2 School of Chemical Engineering and Technology, Collaborative Innovation Center of Chemical Science and Engineering (Tianjin), Tianjin University, Tianjin 300072, China

**Keywords:** guanidyl, synthetic copolymer, nanoparticles, antigen delivery, dendritic cells

## Abstract

Antigen uptake by dendritic cells (DCs) is a key step for initiating antigen-specific T cell immunity. In the present study, novel synthetic polymeric nanoparticles were prepared as antigen delivery vehicles to improve the antigen uptake by DCs. Well-defined cationic and acid-responsive copolymers, monomethoxy poly(ethylene glycol)-*block*-poly(2-(diisopropyl amino) ethyl methacrylate)-*block*-poly(2-(guanidyl) ethyl methacrylate) (mPEG-*b*-PDPA-*b*-PGEM, PEDG) were synthesized by reversible addition-fragmentation chain transfer polymerization of 2-(diisopropylamino)ethyl methacrylate) and N-(*tert*-butoxycarbonyl) amino ethyl methacrylate monomers, followed by deprotection of *tert*-butyl protective groups and guanidinylation of obtained primary amines. ^1^H NMR, ^13^C NMR and GPC results indicated the successful synthesis of well-defined PEDG copolymers. PEDG copolymers could self-assemble into nanoparticles in aqueous solution, which were of cationic surface charges and showed acid-triggered disassembly contributed by PGEM and PDPA moieties, respectively. Significantly, PEDG nanoparticles could effectively condense with negatively charged model antigen ovalbumin (OVA) to form OVA/PEDG nanoparticle formulations with no influence on its secondary and tertiary structures demonstrating by far-UV circular dichroism and UV–vis spectra. *In vitro* antigen cellular uptake by bone marrow DCs (BMDCs) indicated using PEDG nanoparticles as antigen delivery vehicles could significantly improve the antigen uptake efficiency of OVA compared with free OVA or the commercialized Alum adjuvant. Moreover, as the surface cationic charges of OVA/PEDG nanoparticle formulations reduced, the uptake efficiency decreased correspondingly. Collectively, our work suggests that guanidinylated, cationic and acid-responsive PEDG nanoparticles represent a new kind of promising antigen delivery vehicle to DCs and hold great potential to serve as immunoadjuvants in the development of vaccines.

## Introduction

Induction of strong T cell immune responses is one of the major challenges in the development of new vaccines and is also a key to the treatment of diseases induced by insidious pathogens, such as virus, bacteria and parasites, as well as to the immunotherapy of cancers [1–7]. Initiating T cell immunity primarily requires the presentation of antigens by dendritic cells (DCs) [3, 8–12]. DCs are the most effective antigen presenting cells [13], which specialize in capturing antigens, digesting antigens into small peptide fragments, and then presenting them to T cells in the format of their major histocompatibility complex (MHC Class I or II) molecules. Generally, antigens can be divided into two categories according to their source. One is defined as endogenous antigens, which are peptides derived from cytosolic proteins, such as tumor specific antigens synthesized in tumor cells. These antigens are usually loaded onto MHC-I molecules and presented to CD8^+ ^T cells by DCs. The other is called exogenous foreign antigens that are normally processed in endo/lysosomal compartments after internalization by DCs and then loaded onto MHC-II molecules for presentation to CD4^+ ^T cells. Besides, a unique process called cross-presentation for exogenous antigens allows DCs to activate both CD8^+ ^and CD4^+ ^T cells in response to antigens or pathogens [11]. Thus, to evoke strong T cell priming, improving the antigen internalization by DCs should be a feasible and robust strategy.

Recently, antigens loading into micro- or nano-scale particles including aluminum adjuvants [14], PLGA microspheres [8, 15], polyelectrolyte microcapsules [9, 16], polypeptide micelles [17], mesoporous silica nanoparticles [18], gold nanocluster [19–21] and other antigen delivery carriers [22], appears to be an interesting approach for improving antigen presentation by DCs and the immunogenicity of vaccines *in vitro* and *in vivo*. This approach originates from the mimicking of DC’s recognition and internalization of viruses or bacteria that are of a particulate morphology. After subcutaneous or intradermal injection of antigen-encapsulated particles, they would be captured and internalized by DCs and then transported to draining lymph nodes or the spleen, where T cell immunity was evoked. Such nanoparticulate antigen formulations have shown great promise in treating cancers [10, 23]. Depending on the type of antigens and vehicles examined, formulating antigens as a particle can increase the antigen uptake and presentation efficiency by several folds and even more, subsequently activating enhanced T cell responses [10, 17, 24, 25]. Among these carriers, nanoparticles self-assembled by synthetic copolymers have their own unparalleled advantages, such as facile modulation of nanoparticle characters including surface charges, hydrophobicity and decoration of targeting moieties [26, 27], which all have been proved to be crucial parameters for cellular internalization [28] and the initiation of immune responses. Besides, the antigen loading modality such as absorption, encapsulation, conjugation or simple mixing can be easily regulated by adjusting the polymer compositions or by introducing reactive chemical groups to the polymer backbone [14, 17, 29, 30]. Moreover, such nanoparticles are of well reproducibility due to the use of controlled living polymerization techniques for material synthesis and the employment of self-assembly process for formulation preparation.

Herein, we demonstrate a series of synthetic cationic acid-responsive nanoparticles as efficient carriers to deliver antigens into DCs *in vitro*. Monomethoxy poly(ethylene glycol)-*block*-poly(2-(diisopropylamino)ethyl methacrylate)-*block*-poly(2-(guanidyl) ethyl methacrylate) (mPEG-*b*-PDPA-*b*-PGEM, PEDG) copolymers were synthesized by sequential reversible addition-fragmentation chain transfer (RAFT) polymerization, followed by deprotection and guanidinylation reactions. Poly(ethylene glycol) (PEG) was used as the hydrophiphilic block, which has been widely used in nanomedicines and proven to be effective in improving the nanoparticle stability, enhancing endocytosis as well as prolonging nanoparticle circulation time period *in vivo* [31–33]. And very recently, PEG was used to decorate PMMA nanoparticles, which improved the cellular uptake of antigens encapsulated in nanoparticles by DCs and the lymph node targeting *in vivo* [25]. PDPA is a kind of acid-sensitive material with pKa around 6.3, which has been used in constructing pH-responsive drug delivery systems [34, 35]. Guanidinylated polymers used for gene delivery enhanced the gene delivery efficiency with reduced cytotoxicity due to the guanidyl-mediated delocalization of the surface cationic charges and its ability to facilitate nanoparticle endocytosis [36, 37]. So far, copolymers composed of PEG, PDPA and guanidyl and their self-assembled nanoparticles as protein or peptide antigen delivery vehicles have not been reported. In this work, we established a proof-of-concept study of novel guanidinylated and acid-sensitive copolymers as antigen delivery adjuvants. Synthesis and characterization of PEDG copolymers, the self-assembly and acid-responsive property of PEDG nanoparticles were all assessed in detail. By using ovalbumin (OVA) as a model antigen, we demonstrated that antigens formulated in PEDG nanoparticles showed superior uptake efficiency by DCs when compared to soluble antigen or the aluminum adjuvant formulation.

## Materials and experiments

### Materials

Monomethoxy poly(ethylene glycol)-amino (mPEG-NH_2_, average *M*_n_ = 5000 g/mol), 2-(diisopropylamino)ethyl methacrylate (DPA), S-ethylisothiourea hydrobromide, 1,1′-dioctadecyl-3,3,3′,3′-tetramethylindocarbocyanine perchlorate (DiI) and ovalbumin (OVA, grade V) were purchased from Sigma-Aldrich (St. Louis, MO, USA). *N*-(*tert*-Butoxycarbonyl) amino ethyl methacrylate (tBAM) was synthesized as previously described [38]. The macro chain transfer agent mPEG-CTAm was synthesized by the chemical conjugation between mPEG and *S*-1-dodecyl-*S*-(α,α′-dimethyl-α″-acetic acid) trithiocarbonate as described elsewhere [39, 40]. Fluorescein isothiocyanate labeled ovalbumin (OVA-FITC) was provided by Beijing Solarbio Science & Technology Co. (Beijing, China). Alexa Flour^®^ 594 phalloidin and Imject™ Alum Adjuvant were purchased from Thermo Fisher Scientific (Waltham, MA, USA).

### Synthesis of mPEG-*b*-PDPA-*b*-PtBAM

**Figure 1:**
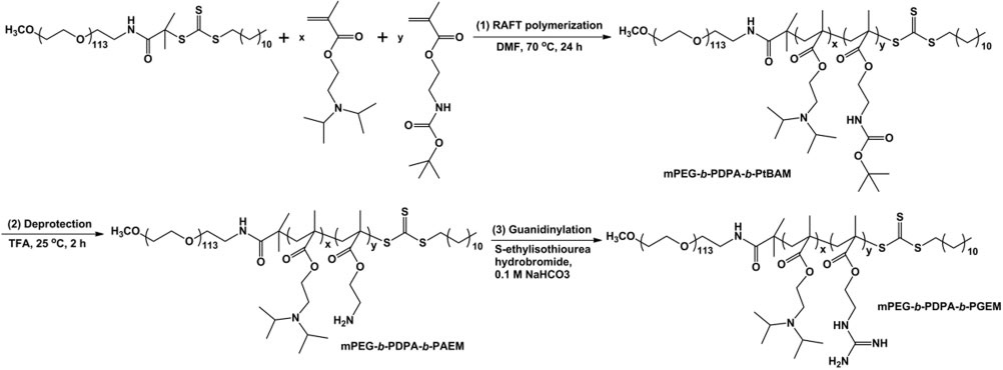
The synthesis route of PEDG copolymers including the sequential polymerization of DPA and tBAM monomers *via* RAFT polymerization, the deprotection of *tert*-butyl groups and the guanidinylation of primary amines.

Monomethoxy poly(ethylene glycol)-*block*-poly(2-(diisopropyl amino) ethyl methacrylate)-*block*-poly(N-(*tert*-butoxycarbonyl) amino ethyl methacrylate) (mPEG-*b*-PDPA-*b*-PtBAM) was synthesized via the sequential RAFT polymerization of DPA and tBAM monomers using mPEG-CTAm as the chain transfer agent. The synthesis route is shown in Figure 1. A typical procedure for mPEG-*b*-PDPA-*b*-PtBAM synthesis was described as follows: mPEG-CTAm (0.1 mmol, 500 mg), DPA (6.5 mmol, 1.39 g) and AIBN (0.02 mmol, 3.0 mg) were added to a Schlenk tube and dissolved in 3 ml anhydrous dimethyl formamide (DMF). The mixture was degassed by three cycles of freeze-pump-thaw and heated at 70°C for 24 h in argon atmosphere. Then, tBAM (3.0 mmol, 653.5 mg) and AIBN (0.02 mmol, 3.0 mg) were added into the tube and the polymerization was continued for another 24 h. The resulted solution was sealed in a dialysis bag with a MWCO of 3.5 kDa, dialyzed against ultra-pure water for 2 days, and then lyophilized to obtain the powder of mPEG-*b*-PDPA-*b*-PtBAM copolymers.

### Synthesis of mPEG-*b*-PDPA-*b*-PGEM

Monomethoxy poly(ethylene glycol)-*block*-poly(2-(diisopropyl amino) ethyl methacrylate)-*block*-poly(2-(guanidyl) ethyl methacrylate) (mPEG-*b*-PDPA-*b*-PGEM, PEDG) was obtained by the deprotection of Boc groups on mPEG-*b*-PDPA-*b*-PtBAM and the following guanidinylation of obtained primary amines. Briefly, mPEG-*b*-PDPA-*b*-PtBAM copolymer (1.0 g) was dissolved in 5 ml of trifluoroacetic acid (TFA) and stirred for 2 h at room temperature. TFA was removed by rotary evaporation and the residue was dissolved in DMF. The resultant solution was then precipitated in diethyl ether and the precipitate was collected by filtration, washed two times by diethyl ether, and dried overnight under vacuum to obtain monomethoxy poly(ethylene glycol)-*block*-poly(2-(diisopropyl amino) ethyl methacrylate)-*block*- poly(2-aminoethyl methacrylate) (mPEG-*b*-PDPA-*b*-PAEM). Finally, the primary amines of mPEG-*b*-PDPA-*b*-PAEM were guanidinylated in 0.1 M NaHCO_3_ solution. After addition of 2 equivalent moles of *S*-ethylisothiourea hydrobromide, the mixture was stirred for 2 days at room temperature. Afterwards, the reaction mixture was placed into a dialysis bag with MWCO of 3.5 kDa and dialyzed against ultra-pure water for 3 days. PEDG copolymers were recovered by lyophilization. PEDG copolymers with different PGEM chain lengths were prepared by adjusting the molar ratio of tBAM monomer at the RAFT polymerization stage.

### Characterization of polymers

^1^H NMR and ^13^C NMR spectrum of PEDG and the intermediate copolymer products were acquired on a Varian Unity spectrometer (500 MHz) using the mixture of D_2_O/phosphoric acid-*d*_3_ as the solvent. The polydispersity index (PDI) of molecular weight was determined by the gel permeation chromatography (GPC) system equipped with a CoMetre 6000 LDI pump, Schambeck SFD GmbH RI2000 refractive index detector and two columns (PLgel 10 μm 10E3A 300 × 7.5 mm and 10E4A 300 × 7.5 mm). The mobile phase was DMF (HPLC grade) containing 0.01 M LiBr and the flow rate is 1 ml/min with a column temperature of 70°C. All samples were filtered through a 0.22-μm filter before analysis and poly(methyl methacrylate) was used as the standard.

### Determination of the critical micelle concentration (CMC) of PEDG copolymer

Pyrene was used as the probe for CMC determination, which was encapsulated into PEDG micelles via a solubilization process. Briefly, a known amount of pyrene in tetrahydrofuran (THF) was added to each of 10 ml vials and then THF was evaporated. 10 ml of copolymer aqueous solutions were added to each vial and equilibrated for 24 h at room temperature. The copolymer concentration varied from 1.0 × 10 ^−^ ^6–1^ mg/ml. The pyrene concentration in copolymer solutions was 6 × 10 ^−^ ^7^ M. The excitation spectra of pyrene were determined by a fluorescence spectrophotometer (Varian Cary Eclipse) at room temperature. The emission wavelength was set at 373 nm with a slit width of 10 nm. The ratio between the fluorescence intensity of excitation wavelength at 337 nm (*I*_337_) and the fluorescence intensity of that at 333 nm (*I*_333_) was calculated and *I*_337_/*I*_333_ values verse logarithmic polymer concentrations was fitted to a sigmoidal curve. The IC50 value was considered as the CMC [41, 42].

### The pH-sensitivity of PEDG nanoparticles

Lipophilic DiI was encapsulated into PEDG nanoparticles via a co-assembly procedure and used as the probe. The pH-sensitivity of PEDG nanoparticles was then examined by monitoring the real-time fluorescence signals of DiI under different pH values. Briefly, 20 mg PEDG and 1 mg DiI were co-dissolved in 2 ml of dimethyl sulfoxide and the solution was sealed in a dialysis bag (MWCO 3.5 kDa), which was dialyzed against PBS buffer (pH = 7.2, 0.01 M) for 2 days. The resulted solution was diluted (the polymer concentration was about 1 mg/ml) and centrifuged (5000 rpm, 10 min) to remove the unencapsulated DiI. The pH of obtained supernatant was adjusted by carefully adding 0.1 M or 0.01 M HCl, which was monitored by a pH meter. The emission spectra of DiI were recorded by a fluorescence spectrophotometer (Varian Cary Eclipse) under the excitation wavelength of 549 nm.

### Preparation and characterization of antigen-absorbed PEDG nanoparticles

Blank PEDG nanoparticles (PEDG NPs) were firstly prepared by the self-assembly of PEDG copolymers in PBS buffer (pH = 7.2, 0.01 M). Briefly, 20 mg PEDG was dissolved in 2 ml of trifluoroethanol and then the solution was added dropwise into 10 ml of PBS buffer under stirring. After evaporation of trifluoroethanol for 24 h and dialysis in water for 2 days, the concentration of PEDG NPs solution was adjusted to 2 mg/ml. To prepare PEDG nanoparticels-formulated OVA vaccines (OVA/PEDG NPs), the solution of PEDG NPs was gently mixed with an equal volume of OVA solution (1 mg/ml) in PBS at 4°C for 30 min. To determine the loading capacity, the obtained mixture was ultra-centrifuged at 100 000*g* for 30 mins, and the amount of unbound OVA in the supernatant and OVA in the precipitate were determined using Pierce^TM^ BCA Protein Assay Kit. Alum adjuvant was also mixed with OVA solution to prepare the OVA/Alum formulation as a positive control.

The size and zeta potential of PEDG NPs or OVA/PEDG NPs were determined by Zetasizer Nano ZS (Malven). And the morphology of nanoparticles was observed by TEM (JEM100CXII, JEOL). Far-UV circular dichroism (CD) was performed to measure the possible changes in protein secondary structure after absorption to nanoparticles. Measurements were collected on a Jasco J-815 (Easton, MD, USA) instrument in the range of 200-280 nm at a protein concentration of 200 μg/ml in PBS. The band width is 1 nm and the scanning speed is 500 nm/min. UV–vis spectra were also recorded to determine the tertiary structure of OVA in the wavelength range of 190–400 nm.

### Isolation and culture of bone marrow dendritic cells (BMDCs)

Mouse BMDCs were prepared according to the previous method [43]. In brief, bone marrow cells were isolated from the femur and tibia of female BALB/C mice (6–8 weeks), and then cultured in RPMI-1640 medium with 10% fetal bovine serum (FBS) and 1% penicillin–streptomycin (Hyclone) supplemented with GM-CSF (20 ng/ml) and IL-4 (10 ng/ml). Six days later, cells were harvested and immature DCs were isolated by flotation through a low-density barrier.

### Antigen uptake by BMDCs and intracellular localization

Immature BMDCs (iDCs) were cultured with free FITC-labeled OVA (OVA-FITC), or that formulated in PEDG NPs or Alum at 37°C for 1 h (OVA concentration, 20 μg/ml). The OVA loading for PEDG nanoparticle formulations was set at 200 μg OVA per 1 mg PEDG. The antigen uptake was analyzed by measuring OVA-FITC positive DCs (CD11C^+^) using flow cytometry (BD LSRFortessa). For intracellular antigen localization, iDCs (1 × 10^6^ cells/ml) were cultured with free OVA-FITC, PEDG NPs or Alum-formulated OVA-FITC at 37°C for 1 h and the antigen dose was 20 μg. Then, cells were fixed with 2% paraformaldehyde in PBS–BSA solution (1% BSA/PBS). Cell membranes were labeled with Alexa Flour^®^ 594 phalloidin for 60 min. Intracellular localization of OVA was examined by confocal laser scanning microscope (CLSM, Leica TCS SP8).

### Statistical analysis

Data are expressed as mean ± standard deviations (SDs). The statistical differences among groups were determined using one-way ANOVA analysis or student's *t*-test in Prism.

## Results and discussion

### Preparation and characterization of PEDG copolymers

**Figure 2:**
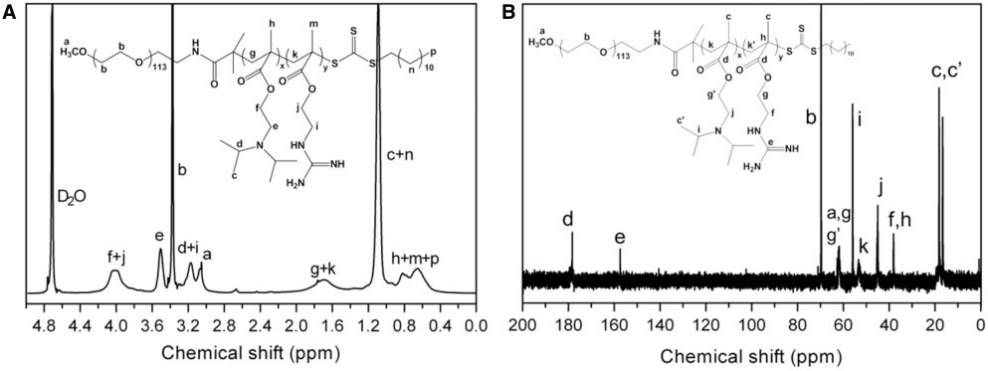
Representative ^1^H NMR and ^13^C MR spectra of PEDG-1 copolymers.

Monomethoxy poly(ethylene glycol)-*block*-poly(2-(diisopropyl amino) ethyl methacrylate)-*block*-poly(2-(guanidyl) ethyl methacrylate) (mPEG-*b*-PDPA-*b*-PGEM, PEDG) copolymers were prepared by a three-step procedure (Figure 1). First, DPA and tBAM monomers were sequentially polymerized using mPEG macroinitiator by RAFT polymerization. ^1^H NMR spectrum (Supplementary data, Figure S1A and B) clearly shows the characteristic chemical shifts assigned to PEG (e.g. hydrogen protons of –(*CH*_2_*CH*_2_)_113_O–at 3.6 ppm), PDPA (e.g. hydrogen protons of –(CH_3_)_2_*CH*N– at 3.0 ppm) and PtBAM (e.g. hydrogen protons of *tert*-butyl –C(*CH*_3_)_2_– at 2.2 ppm) segments, indicating the successful synthesis of mPEG-*b*-PDPA-*b*-PtBAM. The integration areas of these characteristic peaks were used to calculate the polymerization degree of DPA and tBAM monomers. Then *tert*-butyl groups were removed by TFA treatment, and the characteristic peak of hydrogen protons of *tert*-butyl groups at 2.2 ppm disappears from the ^1^H NMR spectrum (Supplementary data, Figure S1C), demonstrating the successful deprotection. Finally, the guanidinylation of primary amines of mPEG-*b*-PDPA-*b*-PAEM was performed using *S*-ethylisothiourea hydrobromide as the guanidinylation agent. In this study, PDPA segments were used to construct the hydrophobic block. The goal of present study was to investigate the antigen delivery by surface guanidinylated nanoparticles, thus, we focused on preparing PEDG copolymers with varying guanidine groups. According to previous studies [35, 44], PDPA with different chain lengths was of similar acid-sensitivity, thus, the polymerization degree for DPA units was set as 60. Figure 2A indicates that all characteristic peaks corresponding to hydrogen protons of PEG, PDPA and PGEM blocks were clearly found, while no impurity peak is observed. Furthermore, only the characteristic peak of carbon atoms of guanidines at 157.2 ppm appears in the ^13^C NMR spectrum (Figure 2B), while that assigned to primary amines was not observed, indicating the guanidinylation of primary amines [45, 46]. GPC results indicate narrow molecular weight distributions of PEDG copolymers according to PDI values (Table 1). The experimental *M*_n_ values of PEDG were revealed to be lower than theoretical ones, due to the interaction between positively charged cationic polymers with columns, which leads to a slower elution [36]. All these results indicate that well-defined triblock PEDG copolymers were successfully obtained. The physicochemical properties of PEDG copolymers are summarized in Table 1.

**Table 1:** The information on chemical structures and molecular weights of PEDG copolymers

Samples	PEG	DP^a^		*M*_n_^a^ (g/mol)	*M*_n_^b^ (g/mol)	PDI^b^
		DPA	GEM			
PEDG-1	5000	60	24	22 688	21 320	1.22
PEDG-2	5000	60	50	27 343	24 376	1.25
PEDG-3	5000	60	76	31 998	27 157	1.19

^a^Determined by ^1^H NMR. DP and *M*_n_ indicate the degree of polymerization and number-average molecular weight, respectively.

^b^*M*_n_ and polydispersity index (PDI) of PEDG copolymers determined by GPC.

### Preparation and characterization of blank PEDG nanoparticles

**Figure 3:**
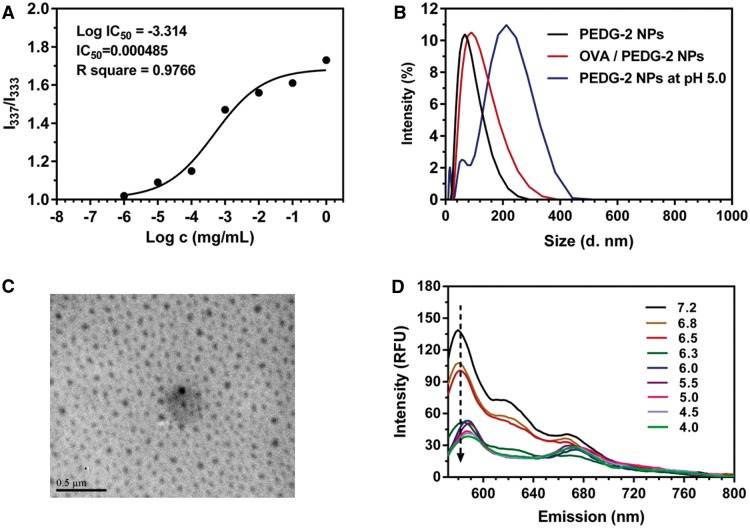
Characterization of the self-assembly of PEDG copolymer in water and the obtained PEDG nanoparticles. (A) The sigmoidal fitting curve of *I*_337_/*I*_33_ ratios as a function of logarithmic polymer concentrations. Pyrene was used as the fluorescence probe. (B) The size of blank PEDG-2 NPs and OVA-loaded PEDG-2 NPs determined by DLS (concentration, 1 mg/ml). (C) A representative TEM image of PEDG-2 NPs and (D) the emission fluorescence profiles of DiI encapsulated in PEDG NPs in environments with different pH values.

**Scheme 1:**
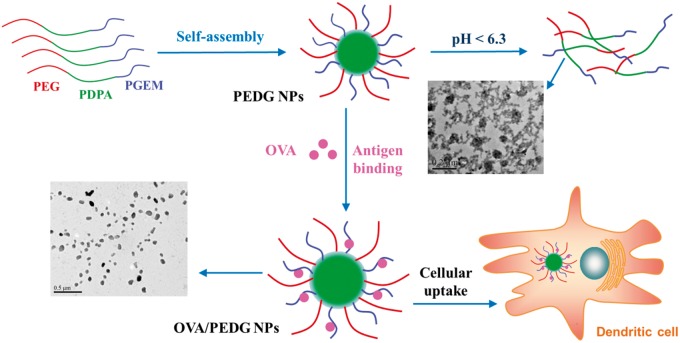
the schematic diagram of self-assembly of PEDG nanoparticles, the acid-triggered nanoparticle swelling or disassembly, the formation of OVA-absorbed PEDG nanoparticles (OVA/PEDG NPs) and the cellular uptake of OVA/PEDG NPs by dendritic cells.

PEDG nanoparticles were formed through the self-assembly of PEDG triblock copolymers in aqueous solution, which is concisely demonstrated in Scheme 1. Pyrene was used as the probe to indicate the formation of micelles. As shown in Figure 3A, the value of *I*_337_/*I*_333_ ratios increased as a function of logarithmic polymer concentrations. Then, *I*_337_/*I*_333_ values were plotted with polymer concentrations and fitted to a sigmoidal curve. The obtained IC50 value was around 4.85 × 10^−^^4^ mg/ml, which was considered as the CMC. Such a low CMC value will ensure the formation of micelles at a polymer concentration of 1 mg/ml. As shown in Figure 3B, PEDG copolymers indeed self-assemble into nanoscale aggregates and TEM image (Figure 3C) indicates a spherical morphology. As shown in Table 2, PEDG nanoparticles are of hydrodynamic sizes under 100 nm and of cationic surface charges resulting from the protonation of guanidine groups in the range of 15–43 mV. In addition, the cationic charges are increased with the increase of PEGM chain length, which is in accordance with the previous study [47, 48]. As a strong base (pK_b _≈_ _0.5), at physiological pH guanidine groups exist in their protonated forms, which are the highly stabilized guanidinium cations.

**Table 2:** Size and zeta potential of blank or OVA-loaded PEDG nanoparticles

Samples		Size (nm)^a^	PDI^a^	ξ (mV)^a^	OVA loading (μg/mg)^b^
Blank PEDG NPs	PEDG-1	57.7 ± 7.3	0.43 ± 0.1	15.21 ± 1.41	—
	PEDG-2	66.0 ± 0.9	0.48 ± 0.01	27.84 ± 1.56	
	PEDG-3	56.6 ± 0.2	0.26 ± 0.007	43.20 ± 1.48	
OVA/PEDG NPs	PEDG-1	128.4 ± 1.7	0.29 ± 0.008	7.26 ± 1.85	210 ± 6
	PEDG-2	87.8 ± 0.97	0.46 ± 0.03	4.51 ± 1.23	640 ± 25
	PEDG-3	53.1 ± 0.05	0.26 ± 0.008	0.75 ± 0.08	682 ± 16

^a^Size and zeta potential (ξ) of PEDG nanoparticles with or without OVA absorption (polymer concentration, 1 mg/ml).

^b^The maximum OVA loading amount was expressed as the mass ratio between OVA and PEDG polymer.

As PEDG contains a pH-sensitive PDPA block (pKa = 6.3), we next examined the nanoparticle′s disassembly profile in different environments. DiI was used as the probe, which was encapsulated into PEDG nanoparticle cores. The real-time emission fluorescence spectrum of DiI was monitored. It was expected that accompanying with the protonation of PDPA, PEDG nanoparticles would swell or disassemble, and meanwhile, the microenvironment of DiI would be partially changed from hydrophobic to hydrophilic, causing a decrease of fluorescence intensity of DiI emission signals. As shown in Figure 3D, when the pH value was reduced from 7.2 to 4.0, the emission band with a maximal peak at around 580 nm gradually decreased. Especially, at pH 6.3, an ∼64% lower of the maximal emission intensity was observed compared with that at pH 7.2, indicating the acid-induced protonation of PDPA segments and the transformation from hydrophobic nature to hydrophilic of PDPA segments. As shown by the TEM image in Scheme 1, a portion of regular spherical nanoparticles disintegrated into random aggregates of polymers and nanoparticles at pH 6.3, and the size (Figure 3B, blue line) also increased, demonstrating the swelling or partial disassembly of nanoparticles. All these results clearly confirm the acid-sensitivity of PEDG nanoparticles.

### Preparation and characterization of antigen-loaded PEDG nanoparticles

It is supposed that OVA proteins can interact with nanoparticles through electrostatic condensing between negative groups on OVA, such as carboxyl, and surface guanidyl of PEDG nanoparticles, to form guanidinium carboxylate salts. The formation of OVA/PEDG nanoparticles (OVA/PEDG NPs) complex was described in Scheme 1. As shown in Table 2, after condensing with OVA antigens, the size of PEDG nanoparticles increased, while the zeta potential remarkably decreased, indicating the coating of OVA on the surface of nanoparticles. For instance, after OVA binding, the average hydrodynamic size of PEDG-2 NPs increased from 66 to 87.8 nm, while the overall surface charges decreased from 27.84 to 4.51 mV. Exceptionally, for PEDG-3 NPs, OVA binding slightly decreases the nanoparticle size, possibly due to the compression of long PEGM chains by OVA protein nanospheres and the formation of stable complex nanoparticles in aqueous solution [36, 37]. The loading amount could reach as high as more than 600 μg OVA per mg polymer for PEDG-2 and PEDG-3 nanoparticles, while that of PEDG-1 nanoparticles is about 200. This imparity is likely due to the less positive charges on PEDG-1 nanoparticles. Just as DNA or RNA condensing with guanidine-modified polymers [36, 37], due to the exposure of cationic PEGM segments, PEDG nanoparticles exhibit high density of surface positive charges, thus, strong electrostatic interaction-mediated antigen condensing were obtained. Besides, PGEM segments can be completely protonated under the physiological pH, and guanidine groups have enhanced affinity as they may form hydrogen bonds with carboxyl of OVA. Therefore, PEDG manifested superior OVA binding capacity.

**Figure 4:**
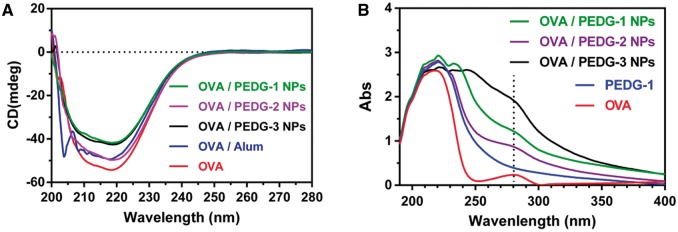
Characterization of the secondary and tertiary structures of free OVA proteins or OVA formulated in PEDG NPs or in Alum adjuvant. (a) Far-UV circular dichroism (CD) and (B) UV–vis spectra.

Then, far-UV CD spectrum was determined for solutions of OVA/PEDG NPs to examine whether the nanoparticle binding with OVA can alter the intrinsic nature of OVA proteins, such as the secondary structures. It has been reported that OVA in aqueous solution exhibits a strong double-negative peak with the 222-nm peak being slightly larger than the peak at 208 nm [21, 49, 50]. The secondary structure of OVA protein was determined by CD to indicate *α*-helices and *α*-helices/β-sheet secondary structures. As shown in Figure 4A, there was no change in the CD spectra comparing free OVA with PEDG nanoparticles-formulated OVA. The spectra of OVA formulated with different PEDG copolymer nanoparticles were also consistent. There are four independent domains and a helical reactive center loop exposed for OVA present in aqueous solution [50, 51]. The tertiary structure was assessed by a UV–vis spectrophotometer. As shown in Figure 4B, OVA shows broad peaks of absorbance at ∼210 and 280 nm and the spectra were consistent across different samples, indicating well preservation of the tertiary structure. All these results demonstrate that PEDG nanoparticle formulations of OVA proteins can effectively maintain their secondary or tertiary structures.

### Cellular uptake and intracellular localization of antigens

**Figure 5:**
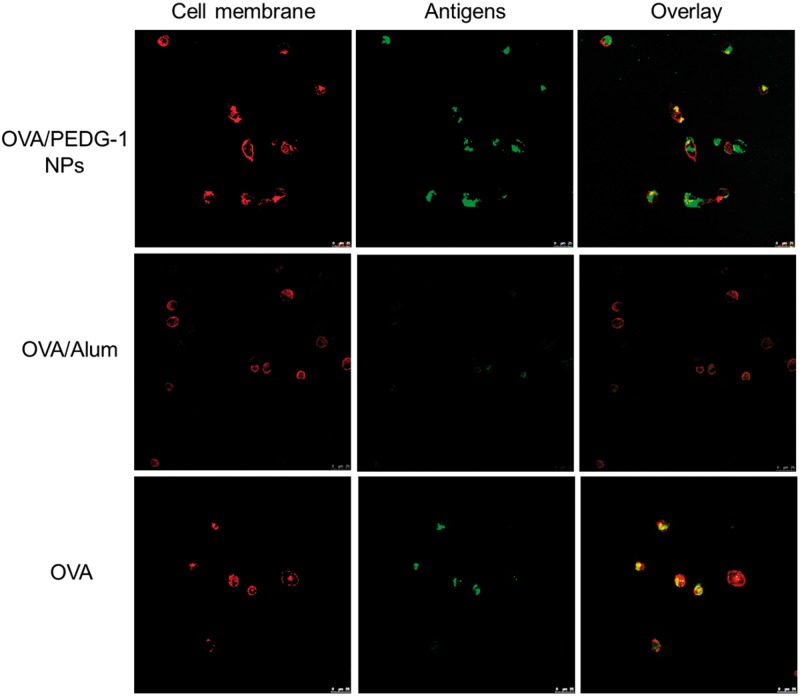
The Effect of PEDG nanoparticles and Alum adjuvant on the cellular uptake and intracellular localization of OVA antigens. BMDCs were cultured with free OVA-FITC, PEDG NPs or Alum adjuvant formulated OVA-FITC at 37 °C for 1 h. The antigen dosage is 20 μg. Representative images of intracellular localization of OVA were captured by CLSM. Cell membranes were labeled with alexa flour^®^ 594 phalloidin.

Antigen uptake and processing by DCs is a crucial step in the activation of T cell immune responses. PEDG nanoparticles can mimic the particulate nature of pathogens, which may facilitate the antigen uptake by DCs. To examine the adjuvant effect of PEDG nanoparticles on antigen internalization, immature BMDCs were incubated with free OVA, or OVA formulated in PEDG nanoparticles or in Alum adjuvant. OVA antigens and cell membranes were labeled by FITC and Alexa Flour^®^ 594 phalloidin, which were indicated by green and red fluorescence signals when observed by CLSM, respectively. As shown in Figure 5, free OVA were mainly localized within cell membrane-derived endo/lysosomes as indicated by the yellow color in the merge image of OVA-FITC and cell membrane signals. And few OVA were also observed in the cytosol. In contrast, OVA delivered by PEDG nanoparticles were mainly localized in the cytosol as shown by the bright green FITC fluorescence signals, while few OVA were also found in cell membrane-derived endo/lysosomes. The representative CLSM images for OVA/PEDG-2 and OVA/PEDG-3 NPs groups were shown in Supplementary data, Figure S2. Moreover, OVA mixed with Alum adjuvant can also be endocytosed by DCs, although the endocytosis amount seems to be low as shown by the weak green fluorescence signal. Representative magnified images of DCs treated by various formulations were shown in Supplementary data, Figure S3.

**Figure 6:**
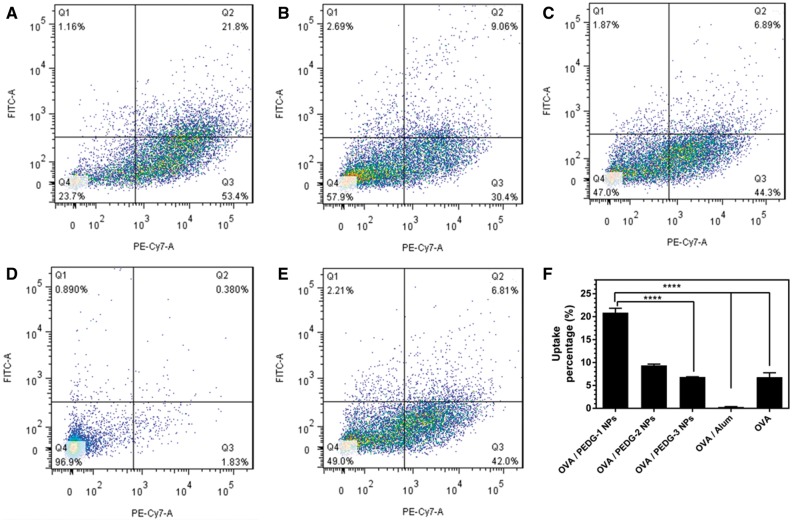
The quantification results of antigen uptake by DCs. FITC and PE-Cy7 was used to label antigens and CD11C^+ ^DCs, respectively. Representative flow cytometry profiles of OVA uptake by BMDCs (A, PEDG-1 NPs; B, PEDG-2 NPs; C, PEDG-3 NPs; D, Alum adjuvant; E, free OVA). (F) The statistical result of the percentage of antigen-internalized DCs in Q2 population determined by flow cytometry.

Antigen uptake was further quantified by flow cytometry. As shown in Figure 6A and 6E (Q2 population), compared with free OVA, PEDG-1 and PEDG-2 NPs shows a significant increase in the percentage of antigen uptake. PEDG-1 NPs can improve OVA antigen uptake to as high as 21.8%. As shown in Figure 6F, OVA uptake of PEDG-1 NPs group was more than 3-fold higher than that of free OVA. Furthermore, PEDG NPs are all more effective in improving OVA uptake than commercialized Alum adjuvant. Besides, as the surface exposed cationic charges of OVA/PEDG nanoparticles decrease, the percent antigen uptake decreases. This is owing to the fact that exposed guanidine groups on the surface of nanoparticles can form hydrogen bonds with the phospholipids or glycoproteins present in the lipid bilayer of cell membranes, facilitating nanoparticle endocytosis and cellular uptake [52]. These data suggest that PEDG nanoparticles effectively facilitated OVA antigen uptake by DCs. It has been reported that free OVA were internalized by BMDCs *via* the mannose receptor-mediated endocytosis since OVA is a mannose-terminated glycoprotein [53]. However, cellular uptake of nanoparticles is usually dominated by clathrin or lipid raft-dependent endocytosis, macropinocytosis or phagocytosis [54, 55]. The enhancement of OVA uptake by DCs might be contributed by the change of endocytosis pathway due to the delivery by PEDG NPs. Besides, the biocompatibility of this kind of cationic nanoparticles should also be noticed because the transmembrane endocytosis of cationic nanoparticles may lead to cell injury (e.g. cell morphology change). In a very recent study [56], the cytocompatibility of one of the copolymer nanoparticles against BMDCs were roughly assessed. The result indicated that at higher concentrations (>80 μg/ml), guanidinylated cationic nanoparticles showed cytotoxicity. To study how the cytotoxicity produced, the cell viability, specific endocytosis pathway and the immunogenicity of these nanoparticles with various positive charges, should be examined. And the immunogenicity of these nanoparticles as well as the immune response of antigen-loaded nanoparticles should also be investigated. Segmental studies are being implemented and other studies would be included in our future works.

## Conclusion

In this study, a series of novel guanidylated copolymers PEDGs were successfully synthesized by RAFT polymerization of DPA and tBAM monomers, followed by the deprotection of *tert*-butyl and guanidination of the primary amines. PEDG copolymers could self-assemble into nanoparticles in PBS. With the increase of PGEM segments, surface charges of PEDG nanoparticles accordingly increased. Under acid environments (pH < 6.3), PEDG nanoparticles would swell or disintegrate into random polymer aggregates. PEDG nanoparticles could effectively complex with OVA antigens with a loading efficiency as high as 600 μg antigen per 1 mg polymer. Far-UV CD and UV–vis spectra indicated similar secondary and tertiary structures comparing free OVA with PEDG nanoparticles-formulated OVA. *In vitro* cellular antigen uptake demonstrated that PEDG nanoparticles could effectively deliver OVA antigens into BMDCs and the antigen uptake efficiency was higher than that of free OVA or Alum adjuvant. In all, PEDG polymeric nanoparticles can efficiently deliver antigens, maintain their essential bioactive structures as well as improve the antigen uptake by DCs. Guanidinylated cationic and acid-responsive PEDG nanoparticles as protein antigen delivery vehicles may open a new window for the development of vaccine adjuvants. Furthermore, the guanidyl numbers in rational design of guanidylated copolymers as potential vaccine adjuvants should be noticed.

## Supplementary data

Supplementary data is available at *REGBIO* online.

## Funding

This work was finally supported by the National Natural Science Foundation of China (81301309, 31670977, 31300732 and 51373199), Natural Science Foundation of Tianjin City (16JCQNJC14200) and Program for Innovative Research Team in Peking Union Medical College.

*Conflict of interest statement. None declared*.
